# *In vivo* rescue of recombinant Zika virus from an infectious cDNA clone and its implications in vaccine development

**DOI:** 10.1038/s41598-020-57545-2

**Published:** 2020-01-16

**Authors:** Gines Ávila-Pérez, Aitor Nogales, Jun-Gyu Park, Desarey Morales Vasquez, David A. Dean, Michael Barravecchia, Daniel R. Perez, Fernando Almazán, Luis Martínez-Sobrido

**Affiliations:** 1 Department of Microbiology and Immunology, University of Rochester Medical Center, 601 Elmwood Avenue, Rochester, New York 14642 USA; 2 Division of Neonatology, Department of Pediatrics, University of Rochester Medical Center, 601 Elmwood Avenue, Rochester, New York 14642 USA; 3 Center for Animal Health Research, INIA-CISA, 28130 Valdeolmos, Madrid Spain; 4 Department of Population Health, Poultry Diagnostic and Research Center, University of Georgia, Georgia, USA; 5 Department of Molecular and Cell Biology, Centro Nacional de Biotecnología (CNB-CSIC), 3 Darwin street, 28049 Madrid, Spain

**Keywords:** Virology, Viral infection

## Abstract

Zika virus (ZIKV) is a mosquito-borne member of the *Flaviviridae* family that has been known to circulate for decades causing mild febrile illness. The more recent ZIKV outbreaks in the Americas and the Caribbean associated with congenital malformations and Guillain-Barré syndrome in adults have placed public health officials in high alert and highlight the significant impact of ZIKV on human health. New technologies to study the biology of ZIKV and to develop more effective prevention options are highly desired. In this study we demonstrate that direct delivery in mice of an infectious ZIKV cDNA clone allows the rescue of recombinant (r)ZIKV *in vivo*. A bacterial artificial chromosome containing the sequence of ZIKV strain Paraiba/2015 under the control of the cytomegalovirus promoter was complexed with a commercial transfection reagent and administrated using different routes in type-I interferon receptor deficient A129 mice. Clinical signs and death associated with ZIKV viremia were observed in mice. The rZIKV recovered from these mice remained fully virulent in a second passage in mice. Interestingly, infectious rZIKV was also recovered after intraperitoneal inoculation of the rZIKV cDNA in the absence of transfection reagent. Further expanding these studies, we demonstrate that a single intraperitoneal inoculation of a cDNA clone encoding an attenuated rZIKV was safe, highly immunogenic, and provided full protection against lethal ZIKV challenge. This novel *in vivo* reverse genetics method is a potentially suitable delivery platform for the study of wild-type and live-attenuated ZIKV devoid of confounding factors typical associated with *in vitro* systems. Moreover, our results open the possibility of employing similar *in vivo* reverse genetic approaches for the generation of other viruses and, therefore, change the way we will use reverse genetics in the future.

## Introduction

Zika virus (ZIKV), a member of the *Flaviviridae* family, became a global public concern because of the correlation of Zika virus epidemic with fetal developmental defects, including highly publicized cases of microcephaly^1^. The viral genome is made of a positive sense, single-stranded RNA molecule (~10.8 kb) that contains a single open reading frame flanked by 5′ and 3′ untranslated regions^2–4^. The viral RNA is translated as a single polyprotein that is co- and post-translationally processed by viral and cellular proteases into three major structural proteins (capsid, pre-membrane and envelope) involved in viral entry, fusion and assembly^5^, and seven non-structural (NS) proteins (NS1, NS2A, NS2B, NS3, NS4A, NS4B and NS5) that are involved in viral RNA replication and transcription, assembly and evasion of the host antiviral responses^6–9^.

Although the majority of ZIKV infections are asymptomatic or associated with a mild febrile illness, infection during pregnancy has been associated with miscarriage and severe congenital malformations, including fetal microcephaly^10,11^, and in adults with the Guillain-Barré syndrome^10,12,13^. ZIKV is primary transmitted to humans through the bite of infected mosquitoes^11^. However, non-vector ZIKV transmission can occur through sexual contact^14,15^ or vertical transmission from infected mothers^16,17^. Currently, there are no approved vaccines or antivirals to prevent or treat ZIKV infection.

The establishment of plasmid-based reverse genetic systems for RNA viruses entails the rescue of recombinant viruses from cDNA clones containing the entire viral genome on a plasmid from transfected culture cells^18^. In 1981, Racaniello and Baltimore provided the basis of this approach with poliovirus^19^. Since then, numerous reverse genetics systems have been developed for positive- and negative-stranded RNA viruses^4,18,20–24^. Plasmid-based reverse genetic approaches allow the rescue of infectious recombinant viruses by the transfection of one or more plasmids encoding the components necessary for the *de novo* generation of infectious virus particles into cultured cells. Importantly, reverse genetic techniques are powerful platforms to modify the viral genome and they have provided critical insights into replication and pathogenesis of multiple viruses^18^. Importantly, viral reverse genetic approaches have also been used as platforms to develop novel and more effective live-attenuated vaccines (LAVs) against viral infections and have significantly contributed to the development of antiviral treatments^18,20^. However, there are intrinsic limitations in the *in vitro* recovery of recombinant viruses using reverse genetic approaches, mostly associated with cell culture-adaptive mutations that might restrict and/or change the phenotype of the virus *in vivo*^25,26^. In the case of LAVs, these mutations could lead to the reversion to a virulent phenotype^27,28^. Likewise, the current need of first generating recombinant viruses in cultured cells rather than directly in validated animal models of infection delays studies aimed to understand and assess viral infections *in vivo*. Therefore, it is of high significance to develop approaches that allow the recovery *in vivo* of infectious recombinant virus from cDNA infectious clones without the current need of a tissue culture step. *In vivo* reverse genetics or recover of virus directly from validated animal models could overcome the concern of virus adaptation to cell cultures and facilitate and simplify the study of the virus and the development of LAVs based on attenuated forms of these viruses.

Recently, we have described a reverse genetic approach for ZIKV based on the use of a bacterial artificial chromosome (BAC), by assembling the full-length genome of ZIKV Rio Grande do Norte Natal (RGN)^27,29^ or Paraiba/2015 strains^30^ under the control of the cytomegalovirus (CMV) immediate-early promoter. Importantly, we demonstrated the feasibility of using this infectious clone to generate a virulent rZIKV with similar *in vitro* and *in vivo* characteristics to the natural Paraiba/2015 isolate^30^. Using the BAC infectious cDNA clone of Paraiba/2015 strain (pBAC-ZIKV), we have explored the possibility to recover infectious rZIKV directly *in vivo* in the type-I interferon (IFN) receptor deficient (IFNAR−/−) A129 mouse model of ZIKV infection^31–33^. The pBAC-ZIKV cDNA clone was complexed with Lipofectamine 2000 (pBAC-ZIKV/LPF) and inoculated in IFNAR−/− A129 mice using different inoculation routes, which are typically used for ZIKV infections. Recovery of rZIKV *in vivo* with high efficiency was observed in mice. In addition, infectious rZIKV was also recovered by direct inoculation of the infectious cDNA clone in the absence of transfection reagent. More Importantly, we demonstrate the feasibility of using this *in vivo* rescue approach with a BAC cDNA infectious clone encoding an attenuated rZIKV (rZIKVatt)^30^ that resulted in sterilizing immunity against aggressive ZIKV challenge. *In vivo* recovery of fully infectious and/or attenuated RNA viruses expands the potential use of reverse genetic systems and open the possibility of developing similar approaches for other viruses, which could be an innovative technology for their study or the future development of LAVs.

## Results

### *In vivo* rescue of rZIKV by inoculation of cells transfected with pBAC-ZIKV

**Figure 1:**
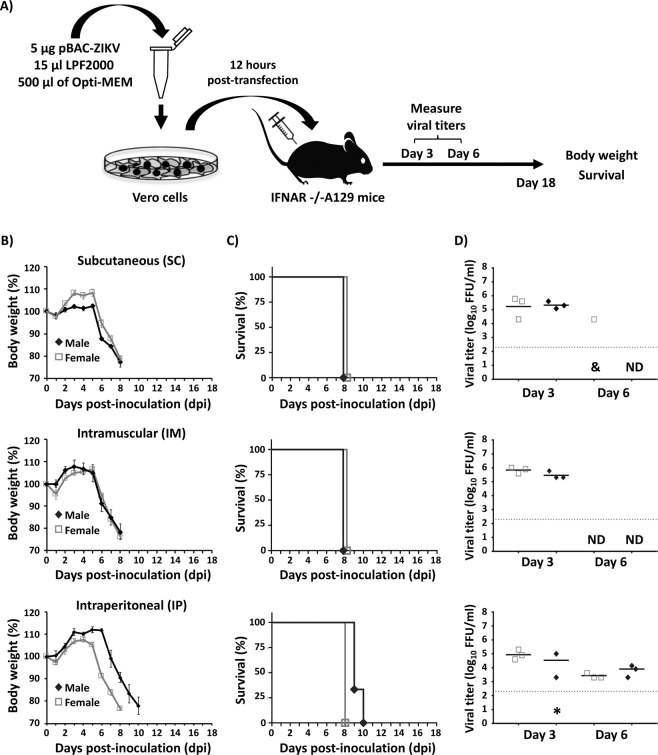
*In vivo* rescue of rZIKV from Vero cells transfected with the BAC cDNA clone. **(A)** Schematic representation of rZIKV *in vivo* rescue approach using transfected Vero cells. (**B–D)** Four-to-five-week-old IFNAR−/− A129 male (N = 3) and female (N = 3) mice were inoculated SC in the footpad (top), IM in the quadriceps muscle (middle) or IP in the peritoneal cavity (bottom) with 1 × 10^6^ Vero cells/mouse transfected with pBAC-ZIKV. Body weight (**B**) and survival (**C**) were evaluated during 18 dpi. Mice that lost more than 20% of their initial body weight or presented hind limb paralysis were humanely euthanized. Error bars represent standard deviations (SD) of the mean for each group of mice. Mice were bled at 3 and 6 dpi and viral titers in sera were determined by immunofocus assay (FFU/ml) (**D**). Symbols represent data from individual mice and bars the geometric means of viral titers. *Virus not detected in one mouse; &, virus not detected in two mice; ND, not detected. Dotted black lines indicate the limit of detection, LoD (200 FFU/ml). No statistically significant differences between the SC, IM and IP routes of inoculation of transfected cells were observed.

Chen *et al*. had previously described a method for the *in vivo* recovery of influenza A virus (IAV), a negative-strand RNA virus, after the inoculation in the nasal cavity of mice with cells transfected with a bacmid containing the entire IAV genome^34^. A similar approach was used to study the potential for reassortment between different IAV subtype strains in ferrets^35,36^. Thus, we conducted similar studies to determine whether this approach was valid for the *in vivo* rescue of rZIKV (Fig. 1**)**. Vero cells (1 × 10^6^ cells, triplicates) were transfected with 5 µg of the pBAC-ZIKV cDNA clone using Lipofectamine 2000 (LPF) (1:3 ratio of cDNA:LPF, herein referred as pBAC-ZIKV/LPF). At 12 h post-transfection (hpt), cells were trypsinized, washed and resuspended in PBS, and used to inoculate mice. Unless otherwise stated, animal studies were performed in 4-5-weeks old, IFNAR−/− A129 mice consisting of 6 mice/group, 3 females and 3 males (Fig. 1A). Mice were inoculated with the pBAC-ZIKV/LPF-transfected Vero cells (1 × 10^6^ cells/mouse) either subcutaneously (SC) in the footpad (Fig. 1B–D, top panels), intramuscularly (IM) in the quadriceps muscle (Fig. 1B–D, middle panels) or intraperitoneally (IP) in the peritoneal cavity (Fig. 1B–D, bottom panels). Animals were subsequently monitored daily for clinical disease signs including tremors, disorientation, hind limb weakness and severe paralysis (data not shown), body weight loss (Fig. 1B) and survival (Fig. 1C) for 18 days post-inoculation (dpi). Independent of the inoculation route, all mice lost weight rapidly starting about 6 dpi (Fig. 1B). Mice showed severe paralysis and succumbed to the disease by 8–10 dpi (Fig. 1C), consistent with effective rZIKV infection^30^. Blood samples were collected at 3 and 6 dpi to test for viremia (Fig. 1D). In general, virus titers in the order ~10^5^ focus-forming units per ml (FFU/ml) were readily observed in samples from 3 dpi (Fig. 1D), in agreement with the morbidity and mortality data (Fig. 1B,C). In addition, successful rescue of rZIKV was similar in both male and female mice (Fig. 1B–D). At 6 dpi, virus was only detected in one female mouse inoculated SC (Fig. 1D, top panel) and in all mice inoculated IP (Fig. 1D, bottom panel). Notably, no virus was detected by 6 dpi in mice inoculated IM (Fig. 1D, middle panel). As an internal control for these experiments, similarly transfected Vero cells were maintained in culture and the rescue of rZIKV was analyzed. Around 10^6^ FFU/ml were detected in all cases by 48 hpt (data not shown), confirming the functionality of the pBAC-ZIKV cDNA clone used for the *in vivo* experiments. Importantly, mice inoculated with Vero cells transfected with empty BAC plasmid did not result in any clinical signs of disease, body weight loss or death; and rZIKV were not detected at any day post-inoculation (data not shown). These data demonstrate that rZIKV can be successfully rescued *in vivo* after inoculation of Vero cells transfected with the pBAC-ZIKV/LPF mixture.

### *In vivo* rescue of rZIKV by direct inoculation of the pBAC-ZIKV/LPF mixture

**Figure 2:**
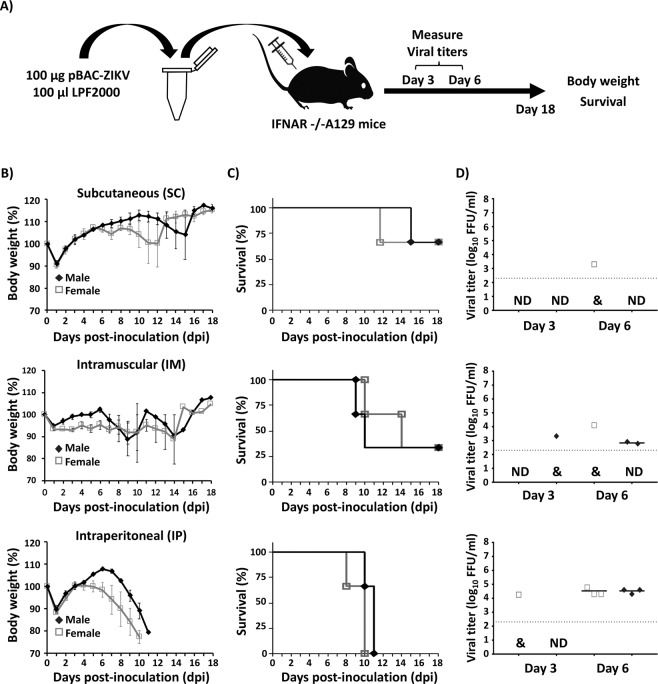
*In vivo* rescue of rZIKV from the pBAC-ZIKV cDNA clone complexed with LPF (pBAC-ZIKV/LPF). **(A)** Schematic representation of ZIKV *in vivo* rescue using pBAC-ZIKV/LPF. (**B–D)** Four-to-five-week-old IFNAR−/− A129 male (N = 3) and female (N = 3) mice were inoculated with the pBAC-ZIKV infectious cDNA clone complexed with LPF SC in the footpad (50 µg/mouse/footpad; total 100 µg, top), IM in the quadriceps muscle (100 µg/mouse, middle), or IP in the peritoneal cavity (100 µg/mouse, bottom). Body weight (**B**) and survival (**C**) were evaluated for 18 days. Mice that lost more than 20% of their initial body weight or presented hind limb paralysis were humanely euthanized. Error bars represent SD of the mean for each group of mice. Mice were bled at 3 and 6 dpi and viral titers in sera were determined by immunofocus assay (FFU/ml) (**D**). Symbols represent data from individual mice and bars the geometric means of viral titers. &, virus not detected in two mice; ND, not detected. Dotted black lines indicate the LoD (200 FFU/ml). Statistically significant differences (p = 0.0026) in survival were observed between mice inoculated IP to those inoculated SC or IM using a Long-rank test. Differences in viral titers were not statistically significant (p = 0.512) at 3 dpi. Differences in viral titers were statistically significant (p = 0.002) at 6 dpi using ANOVA test.

To further determine whether rZIKV could be rescued *in vivo* directly after inoculation of the pBAC-ZIKV cDNA clone, mice were inoculated with 100 µg of pBAC-ZIKV complexed with 100 µl of LPF (1:1 ratio), both diluted in Opti-MEM in a final volume of 200 µl (Fig. 2A). The same three routes described above were evaluated. The group of mice inoculated SC, showed only one female and one male with signs of infection and weight loss from 9 and 12 dpi, respectively (Fig. 2B, top panel). These same mice finally succumbed to the disease by 12 and 16 dpi, respectively (Fig. 2C, top panel). Viremia correlated with the signs of infection, detecting the presence of rZIKV in the serum of the inoculated female mouse at 6 dpi, but not in the male where the signs of infection were delayed (Fig. 2D, top panel). In the IM inoculated group, 2 female and 2 male mice showed evident signs of infection from 7–11 dpi, resulting in rapid weight loss and death (between 7 and 14 dpi; Fig. 2B,C, middle panels). These mice showed viremia at 6 dpi, with the exception of the female where the signs of infection were delayed until 11 dpi (Fig. 2D, middle panels). At 3 dpi, the presence of rZIKV was only detected in one male mouse, although the viremia was prolonged until 6 dpi. Notably, all 6 mice inoculated IP succumbed to viral infection, and rZIKV was detected in the sera of all animals sampled at 6 dpi (Fig. 2B–D, bottom panels). In general, after delivery *in vivo* of the pBAC-ZIKV/LPF mixture, signs of disease were delayed 2 to 3 days compared to mice infected with rZIKV^30^. This delay correlated with the detection of the rZIKV at 6 dpi in mice inoculated with the pBAC-ZIKV/LPF mixture. As internal control for these experiments, Vero cells were transfected with the pBAC-ZIKV/LPF mixture (10 µl containing 5 µg of plasmid DNA) used to inoculate mice resulting in ~2 × 10^6^ FFU/ml of rZIKV by 48 hpt, confirming the quality of the transfection mixture used in the *in vivo* studies (data not shown). Notably, mice inoculated with empty BAC plasmid did not result in the recovery of ZIKV at any day post-inoculation and no clinical signs of disease, body weight loss or death were observed (data not shown). These data demonstrate that rZIKV can be successfully recovered *in vivo* with 100% efficiency after inoculation in the peritoneal cavity of mice with the pBAC-ZIKV/LPF mixture.

### *In vivo* rescue of rZIKV complexed with LPF using different pBAC-ZIKV concentrations

**Figure 3:**
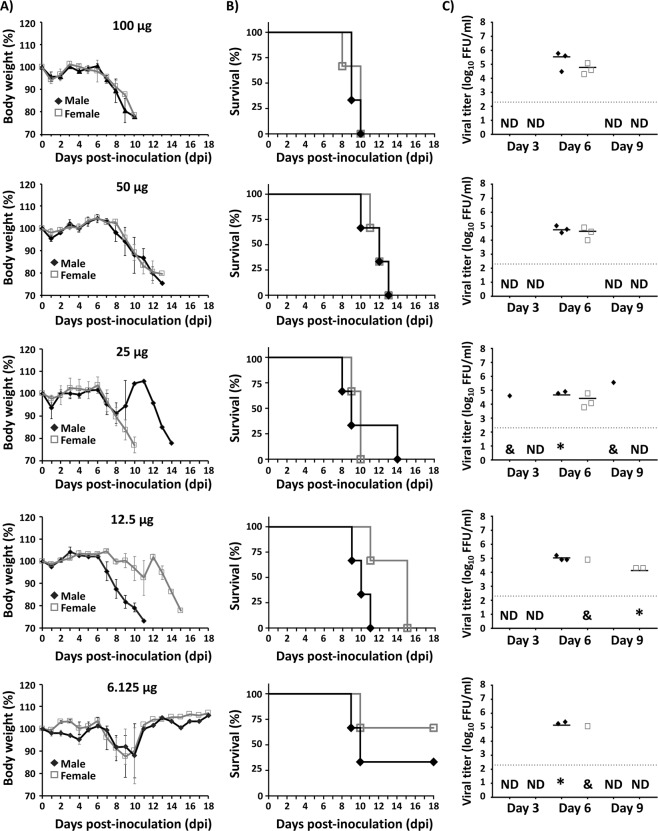
*In vivo* rescue of rZIKV by inoculation of different concentrations of pBAC-ZIKV/LPF. Four-to-five-week-old IFNAR−/− A129 male (N = 3) and female (N = 3) mice were inoculated IP with 100, 50, 25, 12.5 or 6.25 µg (top to bottom) of pBAC-ZIKV complexed with LPF as described in Fig. 2. Body weight loss (**A**) and survival (**B**) were evaluated at the indicated dpi. Mice that lost more than 20% of their initial body weight or presented hind limb paralysis were humanely euthanized. Error bars represent SD of the mean for each group of mice. Mice were bled at 3, 6 and 9 dpi and viral titers in sera (**C**) were determined by immunofocus assay (FFU/ml). Symbols represent data from individual mice and bars the geometric means of viral titers. *Virus not detected in one mouse; &, virus not detected in two mice; ND, not detected. Dotted black lines indicate the LoD (200 FFU/ml). Statistically significant differences (p = 0.008) in survival were observed using a Long-rank test. No statistically significant differences (p = 0.179) in viral titers were observed using ANOVA test.

Since IP inoculation was the most efficient route for the *in vivo* rZIKV rescue, we next evaluated the minimal amount of pBAC-ZIKV/LPF required for such effect (Fig. 3). Groups of mice were inoculated with either 100, 50, 25, 12.5 or 6.25 µg of pBAC-ZIKV cDNA complexed with LPF at a 1:1 DNA:LPF ratio in 200 µl of Opti-MEM. Mice inoculated with 100, 50, 25 and 12.5 µg of the pBAC-ZIKV/LPF mixture lost weight rapidly (Fig. 3A) and all of them succumbed to infection by 8 to 15 dpi, depending on the pBAC-ZIKV concentration (Fig. 3B). The rZIKV was detected in serum at 6 or 9 dpi (Fig. 3C). In animals with delayed body weight loss (one male inoculated with 25 µg and two females inoculated with 12.5 µg of the pBAC-ZIKV/LPF), the virus was detected only at 9 dpi (Fig. 3A–C). On the other hand, only two males and one female inoculated with 6.25 µg of the pBAC-ZIKV/LPF mixture showed body weight loss (Fig. 3A), succumbed to viral infection (Fig. 3B) and presented viremia at 6 dpi. The results with this latter group suggest that 6.25 µg of pBAC-ZIKV is within the lower limit for *in vivo* rescue of rZIKV (Fig. 3C). In general, successful *in vivo* rZIKV rescue was similar in both male and female mice, a finding consistent throughout these studies. Furthermore, these data suggest that recovery of rZIKV *in vivo* is dependent on the concentration of pBAC-ZIKV and as little as 12.5 µg of the pBAC-ZIKV/LPF mixture is sufficient to efficiently rescue rZIKV.

### The rZIKV rescued *in vivo* maintains virulence in a second round of infection

**Figure 4:**
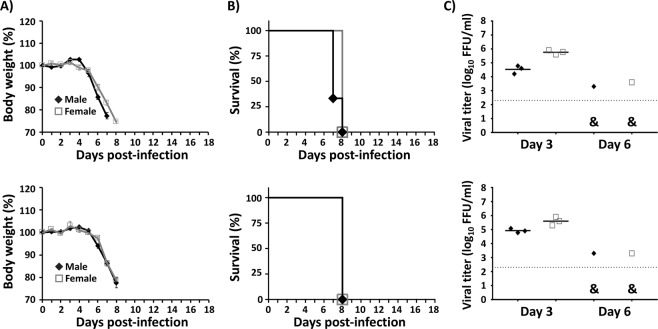
*In vivo* rescued rZIKV is lethal in mice. Four-to-five-week-old IFNAR−/− A129 male (N = 3) and female (N = 3) mice were infected SC in the footpad with 100 FFU/mouse of the rZIKV rescued in male (upper panels) or female (lower panels) mice inoculated with 100 µg of pBAC-ZIKV complexed with LPF (Fig. 3). Body weight loss (**A**) and survival (**B**) were evaluated daily for 18 days post-infection. Mice that loss more than 20% of their body weight or presented hind limb paralysis were humanely euthanized. Error bars represent SD of the mean for each group of mice. At 3 and 6 dpi, mice were bled and viral titers in sera were determined by immunofocus assay (FFU/ml) (**C**). Symbols represent data from individual mice and bars the geometric means of viral titers. &, virus not detected in two mice. Dotted black lines indicate the LoD (200 FFU/ml). No statistically significant differences were observed.

To test whether the rZIKV recovered from the pBAC-ZIKV/LPF-inoculated mice maintained its predicted virulence properties, we performed a second round of infection in mice. Samples of rZIKV isolated from one male and one female mice, previously inoculated with 100 µg of the pBAC-ZIKV/LPF mixture (Fig. 3C), were used to infect naïve mice by the SC route with a lethal dose of the virus (100 FFU/mouse). All mice in the second round of infection lost weight rapidly (Fig. 4A). Signs of ZIKV infection and disease in these mice were indistinguishable from those of similar studies in mice infected with the wild type rZIKV (data not shown) and died by 7 to 8 dpi (Fig. 4B). As expected, high viral titers were detected at 3 dpi, which declined by 6 dpi (Fig. 4C).

### *In vivo* rescue of rZIKV using GenJet transfection reagent or transfection reagent-free naked pBAC-ZIKV cDNA

**Figure 5:**
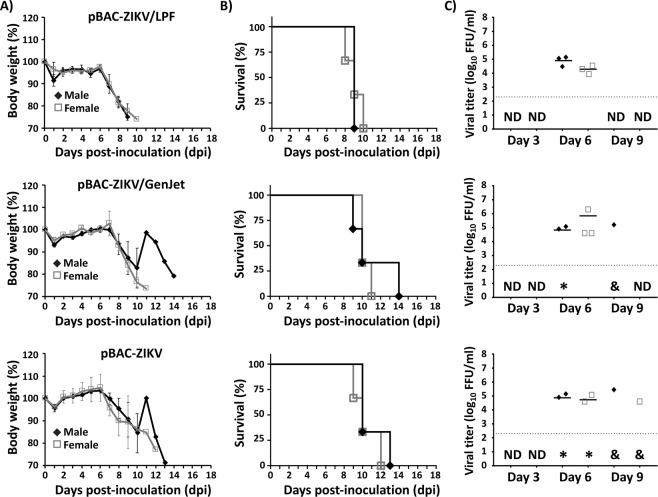
*In vivo* rescue of rZIKV using different transfection methods. Four-to-five-week-old IFNAR−/− A129 male (N = 3) and female (N = 3) mice were inoculated IP with 100 µg/mouse of the pBAC-ZIKV complexed with LPF (top), GenJet (middle) or without transfection reagent (bottom). Body weight (**A**) and survival (**B**) were evaluated during 18 dpi. Mice that lost more than 20% of their initial body weight or presented hind limb paralysis were humanely euthanized. Error bars represent SD of the mean for each group of mice. Mice were bled at 3, 6 and 9 dpi and viral titers in sera were determined by immunofocus assay (FFU/ml) (**C**). Symbols represent data from individual mice and bars the geometric means of viral titers. *Virus not detected in one mouse; &, virus not detected in two mice; ND, not detected. Dotted black lines indicate the LoD (200 FFU/ml). No statistically significant differences were observed among mice inoculated using LPF2000, GenJet or empty BAC.

**Figure 6:**
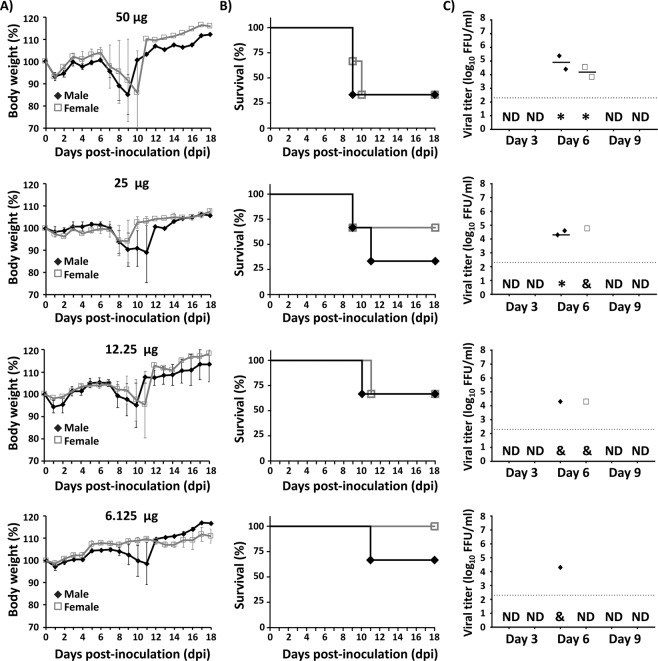
*In vivo* rescue of rZIKV using different concentrations of naked pBAC-ZIKV cDNA. Four-to-five-week-old IFNAR−/− A129 male (N = 3) and female (N = 3) mice were inoculated IP with 50, 25, 12.5, and 6.25 µg of pBAC-ZIKV without a transfection reagent. Body weight (**A**), survival (**B**) and viral titers in mouse sera (**C**) were analyzed as described above. Error bars represent SD of the mean for each group of mice. Symbols represent data from individual mice and bars the geometric means of viral titers. *Virus not detected in one mouse; &, virus not detected in two mice; ND, not detected. Dotted black lines indicate the LoD (200 FFU/ml). Statistically significant differences (p = 0.033) in survival were observed using a Long-rank test. No statistically significant differences (p = 0.336) in viral titers were observed using ANOVA test.

GenJet (SignaGen) is a transfection reagent specifically formulated for *in vivo* DNA delivery. Thus, we further evaluated the *in vivo* efficiency of rescue rZIKV complexed with GenJet instead of LPF. In addition, we also evaluated the rescue efficiency by inoculating mice with naked pBAC-ZIKV cDNA without any transfection reagent (Fig. 5). Mice were inoculated (IP) with 200 µl of a solution containing 100 µg of pBAC-ZIKV complexed with GenJet (1:1 ratio of DNA:GenJet reagent diluted in 10% glucose water solution, pBAC-ZIKV/GenJet) or with 100 µg of transfection reagent-free naked pBAC-ZIKV cDNA (in 200 µl of Opti-MEM). A third group inoculated with the pBAC-ZIKV/LPF mixture was used as control (Fig. 5A–C). As internal controls, Vero cells were transfected *in vitro* with 10 µl of each mixture above (containing 5 µg of plasmid DNA). Viral titers of 2 and 4 × 10^6^ FFU/ml were detected at 48 hpt in the tissue culture supernatants of Vero cells transfected with LPF and GenJet, respectively. As expected, no virus was recovered *in vitro* without the use of a transfection reagent or transfecting empty BAC with any transfection reagent (data not shown). *In vivo*, mice inoculated with either the pBAC-ZIKV/GenJet or the pBAC-ZIKV/LPF mixture began to lose weight by 6–7 dpi (Fig. 5A) and started to succumb to viral infection by 7 dpi (Fig. 5B). In agreement with the morbidity and mortality observations, high viral titers were detected at 6 dpi (Fig. 5A–C, top and middle panels). Only one male inoculated with the pBAC-ZIKV/GenJet mixture showed a slight delay in body weight loss, with viremia at 9 dpi, that succumbed to infection by 14 dpi (Fig. 5A–C, middle panel). No differences were observed between the mice groups inoculated with the pBAC-ZIKV/GenJet or the pBAC-ZIKV/LPF mixtures. Surprisingly, all mice inoculated with 100 µg of transfection reagent-free naked pBAC-ZIKV cDNA showed clear signs of disease (data not shown) and body weight loss (Fig. 5A, bottom panel), and all of them succumbed to viral infection (Fig. 5B, bottom panel). Viremia was detected at 6 and 9 dpi correlating with disease progression (Fig. 5C, bottom panel). Based on these results, we next determined the minimal amount of transfection-reagent free naked pBAC-ZIKV cDNA required for *in vivo* rZIKV rescue (Fig. 6A–C). Mice were inoculated (IP) with either 50, 25, 12.5 or 6.25 µg of naked pBAC-ZIKV cDNA (in 200 µl of Opti-MEM). rZIKV was rescued *in vivo* after inoculation of naked pBAC-ZIKV cDNA and virus recovery was sex-independent with similar efficiencies in female and male mice (Fig. 6A–C). However, recovery of infectious rZIKV using naked pBAC-ZIKV cDNA was less efficient than using LPF or GenJet (Fig. 3A,B). At least 100 µg of the naked pBAC-ZIKV cDNA clone was required (Fig. 5, top panels) compared to 12.5 µg in the pBAC-ZIKV/LPF mixture (Fig. 3**)** for the successful recovery of rZIKV with 100% efficiency.

### *In vivo* rescue of an attenuated rZIKV (rZIKVatt) for vaccine development

**Figure 7:**
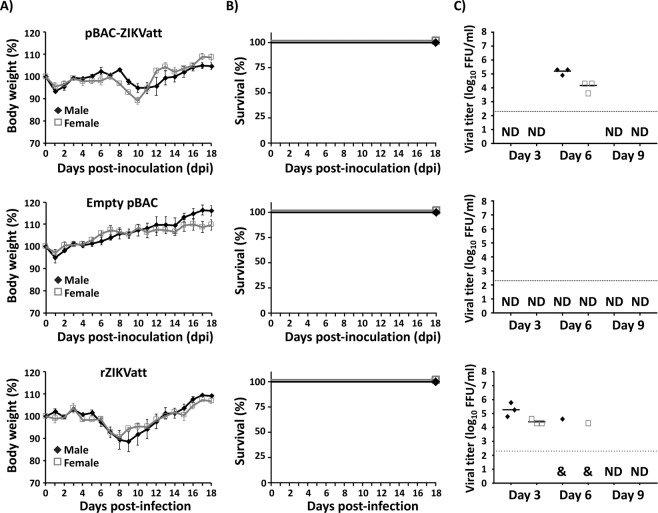
*In vivo* rescue and safety of an attenuated rZIKV (rZIKVatt). Four-to-five-week-old IFNAR−/− A129 male (N = 3) and female (N = 3) mice were inoculated with 100 µg/mouse (top panels) of the pBAC cDNA clone of an attenuated rZIKV (pBAC-ZIKVatt) or with 100 µg/mouse of empty pBAC plasmid (middle panels) both complexed to LPF. As internal control, mice were infected SC in the footpad with a sublethal dose (0.1 X MLD_50_) of the *in vitro* generated rZIKVatt (bottom panels). Body weight (**A**), and survival (**B**) were evaluated at the indicated dpi (top and middle panels) or post-infection with rZIKVatt (bottom panels). Error bars represent SD of the mean for each group of mice. Mice were bled at 3, 6 and 9 dpi or post-infection with rZIKVatt and viral titers in sera were determined by immunofocus assay (FFU/ml) (**C**). Symbols represent data from individual mice and bars the geometric means of viral titers. &, virus not detected in two mice; ND, not detected. Dotted black lines indicate the LoD (200 FFU/ml). No statistically significant differences in survival were observed in mice inoculated with pBAC-ZIKVatt, empty pBAC, or rZIKVatt, using a Long-rank test. Statistically significant differences (p = 0.034) in viral titers were observed in mice inoculated with pBAC-ZIKVatt or rZIKVatt using ANOVA test.

We next explored the feasibility of the *in vivo* reverse genetics system as a potential vaccine platform for the prevention of a typical ZIKV infection (Fig. 7). Towards this end, a ZIKV BAC cDNA clone (pBAC-ZIKVatt) carrying a valine (V) to alanine (A) substitution at position 117 in the viral NS2A protein that renders the virus attenuated in mice compared to the wild type was used^30^. Mice were inoculated (IP) with 100 µg/mouse of the pBAC-ZIKVatt/LPF mixture (Fig. 7A–C, top panels) or with 100 µg of empty pBAC vector/LPF mixture as a control (Fig. 7A–C, middle panels). Another control included mice infected (SC) with a sublethal dose (0.1 X MLD_50_) of the *in vitro* generated rZIKVatt (Fig. 7A–C, bottom panels). As expected, mice inoculated with the empty pBAC cDNA vector/LPF neither lost weight nor die, and virus were undetected (Fig. 7A–C, middle panels). Mice inoculated with the pBAC-ZIKVatt/LPF group lost some weight (~5–10%) between 8 and 10 dpi but were otherwise healthy and survived. Remarkably, viremia was detected in the pBAC-ZIKVatt/LPF group by 6 dpi, indicating successful *in vivo* rescue of rZIKVatt (Fig. 7C, top panel). Similarly, mice infected with the sublethal dose of rZIKVatt lost weight (~10%) between 6 and 8 dpi and showed viremia by 3 dpi, which was prolonged to 6 dpi in some cases, but eventually all mice recovered and survived infection (Fig. 7A,B, bottom panels). As observed previously with the wild type pBAC-ZIKV cDNA clone, signs of disease and viremia were delayed 2 to 3 days after *in vivo* inoculation of the pBAC-ZIKVatt/LPF mixture compared to the direct rZIKVatt inoculation. The internal controls of Vero cells transfected *in vitro* with 5 µg of pBAC-ZIKVatt/LPF used for the *in vivo* rescue resulted in 4 × 10^6^ FFU/ml at 48 hpt, confirming the functionality of the pBAC cDNA clone used for the *in vivo* experiments.

### Immunogenicity and protection efficacy of the rZIKVatt *in vivo* rescue

**Figure 8:**
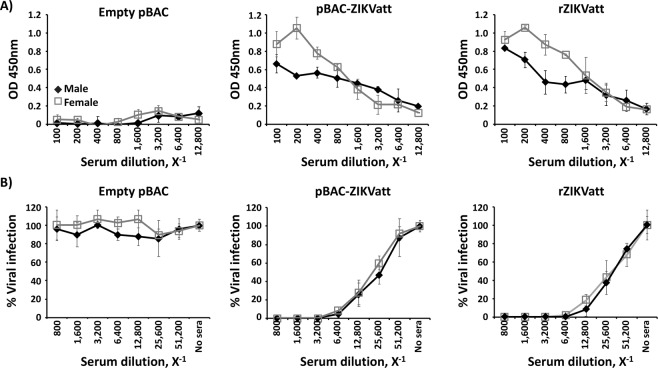
Immunogenicity of *in vivo* inoculation with the pBAC-ZIKVatt cDNA. Mice inoculated as described in Fig. 7 were bled at 21 dpi and the presence of ZIKV antibodies in sera samples were determined by ELISA using cell extracts of ZIKV-infected Vero cells **(A)**. In addition, the presence of ZIKV neutralizing antibodies was evaluated using a FRNT assay on Vero cells **(B)**. Virus in the absence of sera (no sera) was used to calculate 100% viral infection. Error bars represent SD of the mean for each group of sera samples.

**Figure 9:**
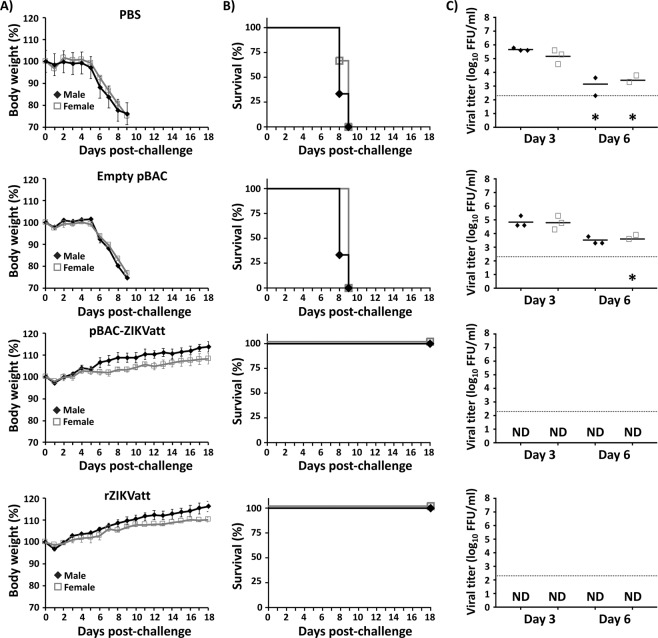
Protection efficacy of the pBAC-ZIKVatt cDNA clone. Mice were inoculated as described in Fig. 7 and at 21 dpi, animals were challenged SC in the footpad with 50 X MLD_50_ of wild type rZIKV. As an internal control, a group of mice were inoculated with PBS. Body weight (**A**) and survival (**B**) were evaluated at the indicated days post-challenge. Mice that lost more than 20% of their initial body weight or presented hind limb paralysis were humanely euthanized. Error bars represent SD of the mean for each group of mice. Mice were bled at days 3 and 6 post-challenge and viral titers in sera were determined by immunofocus assay (FFU/ml) (**C**). Symbols represent data from individual mice and bars the geometric means of viral titers. *Virus not detected in one mouse; ND, not detected. Dotted black lines indicate the LoD (200 FFU/ml). No statistically significant differences in protection efficacy against the lethal challenge with ZIKV were observed in mice inoculated with pBAC-ZIKVatt or rZIKVatt.

We further evaluated whether IP inoculation of the pBAC-ZIKVatt/LPF mixture would lead to protective responses against ZIKV in mice. To that end, mice were inoculated with empty pBAC or pBAC-ZIKVatt complexed with LPF, or infected with a sublethal dose of rZIKVatt as described before. After that, mice were bled at 21 dpi and the sera were collected and evaluated for IgG antibodies against total ZIKV proteins by ELISA (Fig. 8A). Specific antibodies against ZIKV antigens were detected in mice inoculated with pBAC-ZIKVatt/LPF but not in samples from empty pBAC vector/LPF-inoculated mice. Importantly, the levels of antibodies were comparable to those obtained from mice infected with the sublethal dose (0.1 X MLD_50_) of rZIKVatt (Fig. 8A, right panel). Further evaluation of ZIKV neutralizing antibodies (NAb) by focus-reduction neutralization test (FRNT) on Vero cells (Fig. 8B) revealed robust NAb responses in serum samples from mice inoculated with the pBAC-ZIKVatt/LPF mixture, but not in samples from mice inoculated with the empty pBAC vector/LPF control. NAb responses in the pBAC-ZIKVatt/LPF-inoculated group (Fig. 8B, middle panel), were similar to those in serum samples from mice infected with the sublethal dose of rZIKVatt (Fig. 8B, right panel). Given the strong humoral response elicited after inoculation of pBAC-ZIKVatt, we next examined the protection levels of the vaccinated mice against a lethal challenge at 21 dpi with a lethal dose of rZIKV (50 X MLD_50_)^30^. Mice in the pBAC-ZIKVatt/LPF-inoculated group and those in the control rZIKVatt group were fully protected against lethal rZIKV challenge, showing no signs of disease, no body weight loss (Fig. 9A) and viremia below levels of detection. In contrast, mice inoculated with the empty pBAC vector/LPF mixture or PBS mock-inoculated succumbed to viral infection between days 8 and 9 post-challenge (Fig. 9B), with typical clinical signs of ZIKV infection (data not shown) and high virus titers at 3 and 6 days post-challenge (Fig. 9C). Altogether, these results demonstrate that a single IP inoculation of the pBAC-ZIKVatt/LPF mixture is safe, immunogenic and provides sterilizing immunity against lethal challenge with a virulent rZIKV strain.

## Discussion

Reverse genetic approaches have been successfully used in the past to generate recombinant infectious viruses in permissive cells *in vitro*^20–22^. More importantly, reverse genetics has been essential to define the molecular signatures in RNA viruses responsible for replication and transcription, assembly, transmission, host-virus interaction, and pathogenesis^4,18,20–24^. A major caveat with the current use of reverse genetics approaches, however, is that *in vitro* culture systems impose bottlenecks on RNA viruses that eventually leads to selection of a virus population more adapted to grow *in vitro*. Such effects may impede the rescue of biologically relevant viruses, which are ultimately being replaced for those with altered phenotypes *in vivo* compared to the wild-type field isolates. Similar concerns have been raised in the generation of LAVs for the treatment of some viral infections^37–40^. Systems that allow for *de novo* synthesis of RNA viruses directly *in vivo* have the potential to overcome the current limitations of *in vitro* systems, including unwanted mutations, and allow for a more comprehensive analysis of the molecular attributes of field strains in animal models or natural hosts.

In this report, we have expanded the potential of *in vivo* reverse genetics described previously^34^ by showing the efficient rescue of rZIKV from a BAC cDNA clone (pBAC-ZIKV). We systematically analyzed whether Vero cells transfected with pBAC-ZIKV, the pBAC-ZIKV/LPF mixture or naked pBAC-ZIKV without transfection reagent were suitable platforms for generation of rZIKV *in vivo*. We have previously shown that intranasal inoculation of 293T cells transfected with a reverse genetics clone of IAV in a bacmid DNA leads to generation of infectious virus^34^. Here we show that other routes (SC, IP, IM) are also suitable for transfection-based inoculation *in vivo* reverse genetics, at least for the rescue of rZIKV. On the other hand, we developed an *in vivo* rescue approach based on the direct inoculation of the pBAC-ZIKV complexed with LPF. Direct inoculation by either the SC, IM, IP routes of pBAC-ZIKV/LPF resulted in successful and efficient rZIKV recovery, with the IP route being the most consistent and efficient. While DNA without any transfection reagent has been shown to be effective for gene transfer and expression following direct injection into skeletal muscle or skin *in vivo*, this is the first report of IP delivery and expression of naked DNA. Regardless of the inoculation route used, virus produced using the *in vivo* reverse genetics approach reproduced disease associated with ZIKV infection in the mouse model, including neurological signs, body weight loss, and death. We observed that ZIKV infection was delayed 2–3 days after pBAC-ZIKV inoculation compared to ZIKV-infected mice, which can be explained by the fact that the viral RNA has to be transcribed from the pBAC-ZIKV by the cellular RNA polymerase II in the nucleus of transfected cells and further amplified in the cytoplasm by the viral replicase. Considering the time needed to rescue viruses using reverse genetics in cultured cells, our *in vivo* reverse genetics also represents a significant improvement in the time that viruses can be studied in animal models of infection. The rZIKV generated after *in vivo* inoculation conserved the infectivity and virulence when used to infect new mice, which is important because it implies that the system recapitulates a bona fide ZIKV replication cycle. Further studies suggest that *in vivo* ZIKV rescue is dependent on the concentration of plasmid, but the virus can be recovered even in the absence of transfection reagent, albeit with lower efficiency. This also represent an advantage over current *in vitro* reverse genetic approaches that requires the use of expensive transfection reagents for successful recovery of viruses in cultured cells. Overall, these results demonstrate the feasibility of using the *in vivo* reverse genetics approach for the successful rescue of rZIKV *in vivo*. This methodology could be useful to directly test the contribution of specific mutations in the viral genome *in vivo*, without the need of prior recovering and amplifying the virus *in vitro*, to minimize potential mutations associated with passaging the virus in culture cells and overall reducing the time required to study viruses in validated animal models of infection. Moreover, this approach could be useful to rescue other RNA viruses, with poor replication properties in cultured cells^41^.

It is important to indicate that our studies are based on the use of IFNAR −/− A129 mice that are deficient in type I IFN responses. In this animal model, infection with ZIKV using different routes (e.g. subcutaneous, intraperitoneal or intramuscular) results in neurological disease and the animals succumb to viral infection with high viral load in the blood and organs (liver, spleen, kidney, brain, and spin cord), consistent with ZIKV infection and manifestation in humans. Contrary, infection of WT mice with ZIKV does not results in clinical signs of infection, mortality or detection of the virus at different times post-infection in the blood or organs of infected animals^33,45^. Thus, the use of IFNAR −/− A129 immunodeficient mice represented the best suitable model to demonstrate the successful rescue of recombinant ZIKV using *in vivo* reverse genetics. Further studies using a more relevant animal model of ZIKV infection, currently outside the scope of this manuscript, will demonstrate the feasibility of using similar *in vivo* reverse genetics for the feasibility to generate recombinant ZIKV.

Previous studies have reported that *in vitro* transcribed Hepatitis C virus (HCV) viral (v)RNA was infectious after direct injection into the liver of chimpanzees^42,43^. This was an important technical advance in the field, since HCV does not replicate efficiently in cell cultures^25,44^. However, it also has some major limitations, including the need of transcribing the vRNA before *in vivo* delivery, which may introduce spurious mutations and is also limited to the quality, quantity, and stability of the transcribed vRNA *in vivo*. In that sense, our approach to recover recombinant viruses *in vivo* represents a major advance since plasmid DNA is easier to produce and more stable that vRNA transcripts. The plasmid DNA was inoculated IP, which allows for systemic delivery and therefore no specific organs have to be injected. We were able to obtain 100% viral rescue success when using up to 12.5 μg of plasmid DNA, alone or in combination with transfection reagents. Also, our results demonstrate that the IM route, a more validated *in vivo* DNA delivery, also serves as a useful method for *in vivo* rescue of rZIKV, although the IP route was more efficient in the recovery of rZIKV, challenging the gold standard (IM route) of plasmid DNA delivery *in vivo*.

DNA vaccines have several advantages over LAVs, including stability and cost^45,46^. However, DNA vaccines have been hampered by issues related to poor immunogenicity and/or the need for inoculation of high amounts (mgs) in order to generate adequate protective responses. In contrast, LAVs have been shown to provide efficient, multidimensional protective responses by stimulating humoral and cellular protective immune responses. The major drawback of LAVs is that they may require specialized culture system substrates for production and a cold-chain to maintain the integrity of the vaccine^45,47^. The ability to combine the advantages of these two systems, like the one described in this report, opens the exciting possibility for greatly improving vaccines and vaccination strategies for a myriad of pathogens, including ZIKV. We took advantage of our previously described pBAC-ZIKVatt cDNA infectious clone^30^ to show that rZIKVatt was efficiently recovered and safe *in vivo*, which in turn produced strong humoral responses, including high levels of neutralizing antibodies capable of protecting mice against a lethal rZIKV challenge. This new *in vivo* viral rescue platform for vaccine development offers numerous advantages compared to traditional approaches and combines the advantages of DNA vaccines (stability, easy production, low manufacturing costs and limited cold-chain requirements) with the strengths of LAVs (dose sparing, broad innate and adaptive immune responses)^48–51^. More importantly, our new *in vivo* reverse genetics approach could be extended to vaccine development for other RNA viruses that represent a concern in human health.

## Material and Methods

### Cells

African green monkey kidney epithelial Vero cells (ATCC, CCL-81) were grown in Dulbecco’s modified Eagle’s medium (DMEM; Mediatech, Inc) supplemented with 5% fetal bovine serum (FBS), 2 mM L-glutamine, 100 U/ml penicillin and 100 μg/ml streptomycin at 37 °C with 5% CO_2_.

### ZIKV and infectious BAC cDNA clones

The rZIKV wild type Paraiba/2015 and its corresponding attenuated (att) strain were previously described^30^ and obtained after transfection of Vero cells with the corresponding infectious BAC cDNA clones. The rZIKVatt contains a valine (V) to alanine (A) substitution at position 117 in the viral NS2A protein that renders the virus attenuated in mice^30^. Briefly, ZIKV genomes were assembled in the BAC plasmid pBeloBac11 under the control of the CMV promoter and flanked at the 3′ end by the hepatitis delta virus (HDV) ribozyme and the bovine growth hormone (BGH) termination and polyadenylation sequences^52^. Infectious clones were propagated in the DH10B strain of *E. Coli* (Gibco/BRL), and BAC were prepared using the Qiagen plasmid Giga kit, following the manufacture specifications.

### Recovery of rZIKV from BAC cDNA clones in Vero cells

Vero cells grown to 90% of confluence (6-well plate format, 1 × 10^6^ cells/well, triplicates) were transfected with 5 μg of the ZIKV BAC cDNA clones using 15 μl of LPF (Invitrogen), as described previously^29^. At 6 hpt, media was removed and replaced with grown media supplemented with 2% FBS. For *in vitro* rescues, tissue culture supernatants were collected after 3–4 days, when cytopathic effect (CPE) was apparent (60–80%), and stored at −80 °C. Virus stocks were propagated in Vero cells and titrated by immunofocus assay in Vero cells using the pan-flavivirus envelope (E) protein monoclonal antibody (mAb) 4G2 from BEI Resources (NR-50327)^27^. Viral titers were expressed as focus-forming units per ml (FFU/ml)^27^.

### Mouse studies

IFNAR −/− A129 mice (Jackson Laboratory) were bred and maintained in the animal care facility of the University of Rochester Medical Center (URMC). All mice were housed under specific pathogen-free conditions. Mouse experiments were approved by the University of Rochester Committee of Animal Resources and carried out in compliance with the recommendations in the Guide for the Care and Use of Laboratory Animals of the National Research Council^53^. Four-to-five-week-old female and/or male mice were anesthetized (IP) with a solution of ketamine (100 mg/kg) and xylazine (10 mg/kg), and then inoculated in the footpad (subcutaneous, SC), in the quadriceps muscle (intramuscularly, IM) or in the peritoneal cavity (intraperitoneally, IP) as indicated in the main text. Morbidity (body weight loss and paralysis) and mortality were monitored for 18 days. Mice showing severe signs of paralysis or mice that lost more than 20% of their initial body weight were humanely euthanized following AVMA guidelines. Statistical analyses were performed using PRISM software (GraphPad, version 7).

### Determination of viremia in sera samples

Mouse sera were collected by submandibular bleeding at the indicated dpi and blood was maintained at room temperature for 30 min to allow clotting. Serum was separated by centrifugation at 5,000 rpm for 20 min and immediately stored at −80 °C. Viral titers were determined by FFU assays in Vero cells^27^.

### Enzyme-linked immunosorbent assay (ELISA)

To assess the levels of virus-specific antibodies present in the sera of vaccinated mice, ELISAs were performed as previously described^27,54,55^. Briefly, 96-well ELISA plates (ThermoFisher) were coated with extracts from ZIKV-infected Vero cells, and incubated overnight at 4 °C. Coated wells were blocked with 1% BSA in PBS, and incubated with 2-fold serial dilutions of mice sera (starting dilution of 1:100) for 1 h at 37 °C, following by incubation with horseradish peroxidase (HRP)-conjugated goat anti-mouse IgG for 1 h at 37 °C. Reactions were developed with tetramethylbenzidine (TMB) substrate (BioLegend) for 10 min at room temperature, quenched with 2 N H_2_SO_4_ and read at 450 nm in a Vmax Kinetic microplate reader (Molecular Devices).

### Focus-reduction neutralization test (FRNT)

The presence of neutralizing antibodies in the sera of vaccinated mice were determined by FRNT as previously described^56^. Briefly, 2-fold serial dilutions of sera (starting dilution of 1:800) were incubated with 25 FFU of rZIKV for 3 h at 37 °C, and used to infect subconfluent (80%) monolayers of Vero cells in 96-well plates. After 90 min of viral absorption at 37 °C, virus-sera mixture was removed, and the cells overlaid with 100 μl of growth medium containing 2% FBS and 1% Avicel. Cells were fixed at 36 h post-infection with 4% paraformaldehyde and prepared for immunostaining assay using 1 μg/ml of mAb 4G2. Viral plaques were visualized using Vectastin ABC kit and DAB HRP substrate (Vector Laboratories Inc.), following the manufacturer’s instructions.
